# Localized subacute thyroiditis presenting as a painful hot nodule

**DOI:** 10.1186/1472-6823-14-4

**Published:** 2014-01-08

**Authors:** Lian-Xi Li, Xing Wu, Bing Hu, Hui-Zhen Zhang, Han-Kui Lu

**Affiliations:** 1 Department of Endocrinology and Metabolism, Shanghai Diabetes Institute; Shanghai Clinical Center for Diabetes; Shanghai key Laboratory of Diabetes Mellitus, Shanghai Jiao Tong University Affiliated Sixth People’s Hospital, 600 Yishan Road, Shanghai 200233, China; 2 Department of Ultrasonography, Shanghai Jiao Tong University Affiliated Sixth People’s Hospital, 600 Yishan Road, Shanghai 200233, China; 3 Department of Pathology, Shanghai Jiao Tong University Affiliated Sixth People’s Hospital, 600 Yishan Road, Shanghai 200233, China; 4 Department of Nuclear Medicine, Shanghai Jiao Tong University Affiliated Sixth People’s Hospital, 600 Yishan Road, Shanghai 200233, China

**Keywords:** Subacute thyroiditis, Thyroid nodule, Hot nodule

## Abstract

**Background:**

A diagnosis of subacute thyroiditis is readily considered when patients present with a particular set of typical clinical characteristics. Subacute thyroiditis sometimes presents as a solitary cold nodule; however, the presence of a hot nodule in patients with subacute thyroiditis is exceedingly rare.

**Case presentation:**

Here, the case of a 57-year-old woman complaining of pain in the left neck and fatigue for two weeks is presented. Physical examination revealed a painful and tender nodule with a diameter of approximately 1.5 cm in the left neck, although all laboratory tests, including white blood cell count, neutrophil percentage, erythrocyte sedimentation rate (ESR), thyroid function, and thyroglobin levels, were normal. A neck ultrasound revealed a hypoechoic mass (1.5 × 0.8 cm) in the left thyroid, and thyroid scintigraphy of the left thyroid with Technetium-99 m (99 m-Tc) demonstrated a focal accumulation of radiotracer. Furthermore, fine-needle aspiration biopsy from the nodule revealed the presence of multinuclear giant cells. The patient was well; there was no cervical mass detected upon palpation following two months of prednisone treatment, and follow-up ultrasound screening and scintigraphy demonstrated the disappearance of the nodule.

**Conclusion:**

This case, presenting with a localized painful hot nodule, normal thyroid function, normal ESR, and normal serum thyroglobulin levels, is a rare case of subacute thyroiditis, which should be considered during differential diagnosis.

## Background

Subacute thyroiditis, also known as de Quervain’s thyroiditis, giant-cell thyroiditis, or subacute granulomatous thyroiditis, is a spontaneously remitting inflammatory disease of the thyroid gland [1,2]. Subacute thyroiditis is generally caused by viral infection and is the most common cause of a painful thyroid [1,3]. Patients with subacute thyroiditis usually have a history of antecedent viral infection and subsequently suffer from neck pain, thyroid tenderness, fever, and fatigue. Upon physical examination, the thyroid of the patient is often tender and diffusely enlarged.

In most cases, a diagnosis of subacute thyroiditis is usually self-evident and can be made based on patient history, physical and laboratory findings, and the clinical course of the disease. In some cases, in addition to the clinical course and features, fine needle aspiration cytology, ultrasound, and scintigraphy analyses may support the diagnosis of subacute thyroiditis. For example, thyroid radioisotope scanning generally demonstrates a low uptake of Technetium-99 m (99 m-Tc) or 131I [4]. However, patients with subacute thyroiditis sometimes present with puzzling clinical features that can escape early recognition [2,3,5,6]. Here, a patient with subacute thyroiditis, who presented with a solitary painful thyroid nodule in the absence of typical laboratory test characteristics that would suggest subacute thyroiditis and whose 99 m-Tc thyroid scan revealed a hot nodule in the left lobe of thyroid, is described. To the best of our knowledge, the presence of a hot nodule in a patient with subacute thyroiditis has not been previously reported.

## Case presentation

A 57-year-old woman with no history of thyroid disease visited our outpatient endocrine clinic on July 27, 2012. Two weeks prior, she had developed symptoms of pain in the left neck and fatigue. Physical examination revealed a focal nodule of the left thyroid lobe that had a diameter of approximately 1.5 cm without local redness or lymph node enlargement, which was painful and tender upon examination. There was no fever or signs of hyperthyroidism such as tachycardia, insomnia, or tremors. The patient’s thyroid function tests were normal (thyroid stimulating hormone = 1.25 mU/L, normal range: 0.27-4.2 mU/L; free triiodothyronine = 5.59 pmmol/L, normal range: 3.1-6.8 pmmol/L; free thyroxine = 18.80 pmmol/L, normal range: 12–22 pmmol/L) and tests for anti-thyroglobulin (<10 KIU/L, normal range: 0–115 KIU/L), anti-thyroid peroxidase (10.54 KIU/L, normal range: 0–35 KIU/L), and anti-thyrotropin-receptor antibodies (<1 U/L, normal range: 0–2 U/L) were negative. The patient’s serum thyroglobulin levels were normal (61.32 ug/L, normal range: 1.40-78 ug/L); her white cell count was 5.2 × 10^9^/L with a normal differential, and her erythrocyte sedimentation rate (ESR) was 20 mm/hour (normal range: 0–38 mm/h).

**Figure 1:**
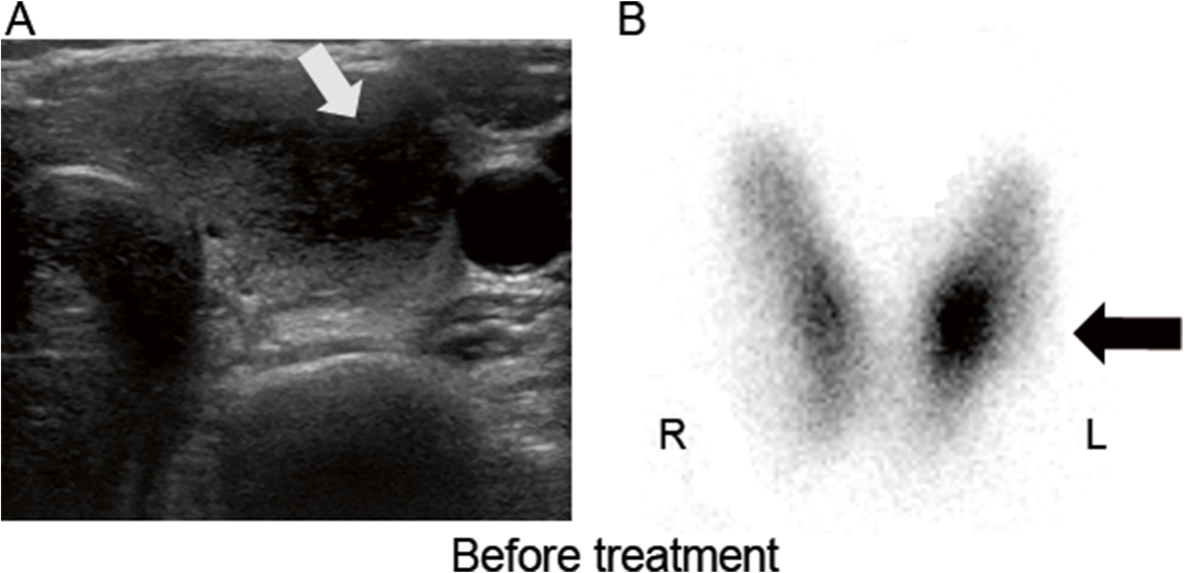
**Initial thyroid ultrasound scanning and 99 m-Technetium scintiscan. A**. Thyroid ultrasonography revealed a dyshomogeneous and hypoechoic nodule (15 × 0.8 mm) with an irregular and poorly defined border in the left thyroid lobe (indicated by a white arrow). **B**. Thyroid scintigraphy with 99 m-Tc showed a focal accumulation of radiotracer uptake in the lower lobe of the left thyroid, which represents the palpable tender nodule (indicated by a black arrow).

**Figure 2:**
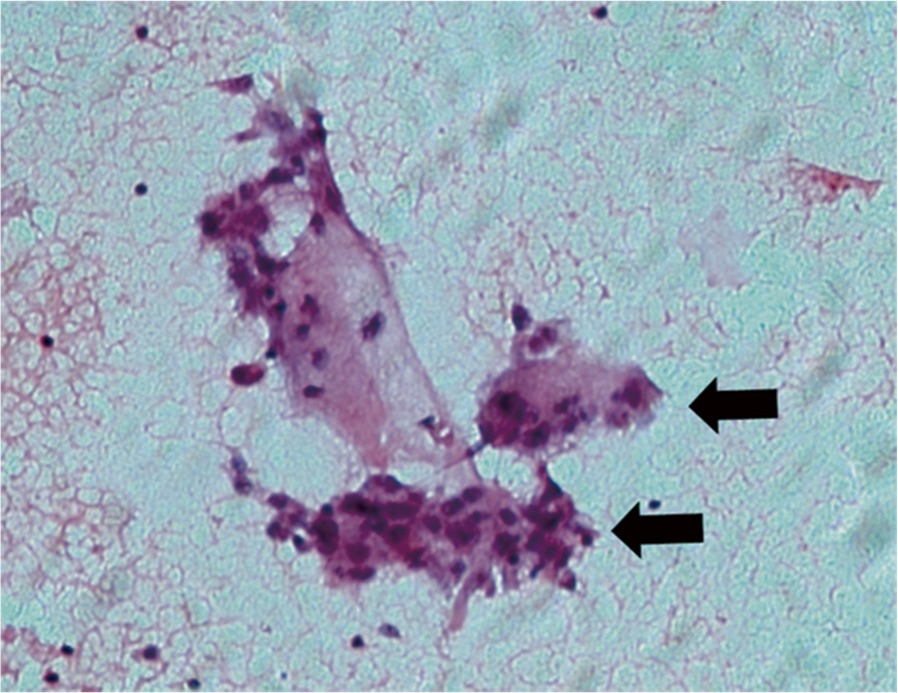
**Fine-needle aspiration cytology of the left thyroid nodule.** Fine-needle aspiration biopsy from the nodule in the left lobe revealed multinuclear giant cells in the thyroid nodule consistent with subacute thyroiditis (indicated by black arrows).

**Figure 3:**
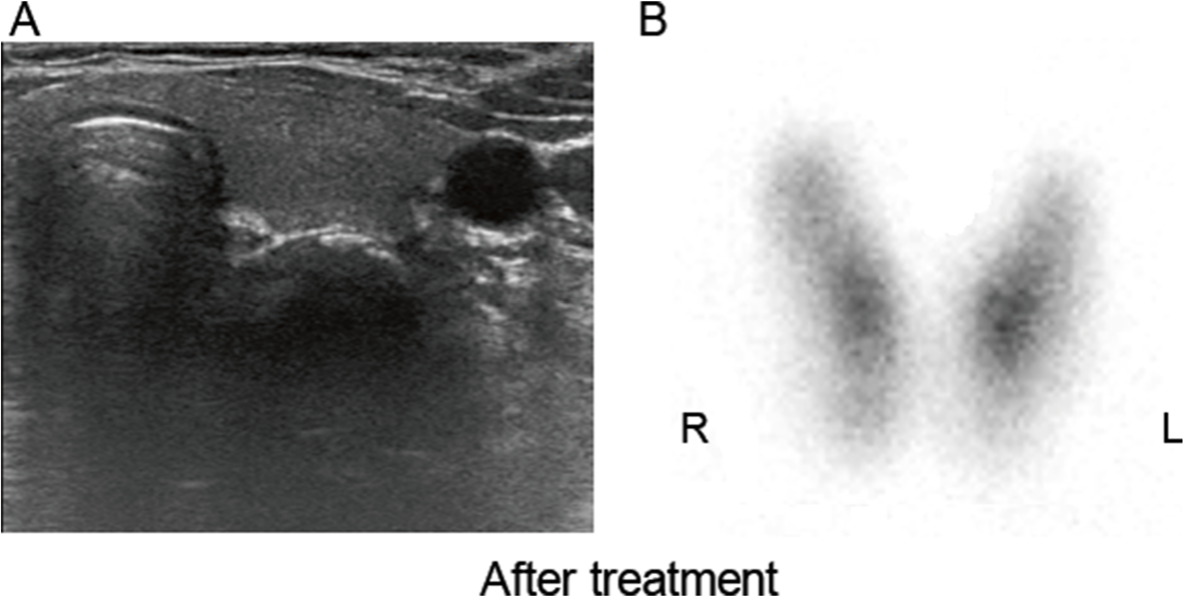
**Follow-up ultrasound scanning and 99 m-Technetium scintiscan. A**. Follow-up ultrasound scanning demonstrated the disappearance of the hypoechoic nodule in the left thyroid lobe. **B**. Follow-up scintigraphy showed the disappearance of the “hot” nodule in the left thyroid lobe and an even distribution of radionuclide in both lobes.

A thyroid ultrasound examination revealed a dyshomogeneous and hypoechoic mass (1.5 × 0.8 cm) in the left thyroid lobe that exhibited an irregular and poorly defined border (Figure 1A). Thyroid scintigraphy with 99 m-Tc demonstrated a focal accumulation of radiotracer uptake in the lower part of the left thyroid lobe but a normal uptake and configuration of the middle and upper portion of the left lobe and the right lobe (Figure 1B). Fine-needle aspiration biopsy from the nodule in the lower left lobe revealed multinuclear giant cells consistent with subacute thyroiditis (Figure 2). Subsequently, localized subacute thyroiditis was suspected and the patient was put on prednisone (30 mg/day) for 10 days, which resulted in a rapid resolution of the neck pain. Within a week, the patient’s fatigue had disappeared and the tender thyroid nodule had regressed. Prednisone was gradually withdrawn and ultimately stopped after 2 months at which time palpation did not demonstrate a cervical mass. Repeated ultrasound screening revealed a disappearance of the hypoechoic nodule (Figure 3A) and follow-up scintigraphy analysis found the thyroid exhibited an even distribution of radionuclide in both lobes (Figure 3B).

## Conclusions

Subacute thyroiditis is the most common cause of non-autoimmune thyroiditis [7]. In addition to the typical clinical signs, characteristic ultrasound findings of subacute thyroiditis include the presence of an ill-defined hypoechoic area with a nonhomogeneous pattern [8]. Recently, Ruchala et al. [9] demonstrated the usefulness of sonoelastography for the diagnosis of subacute thyroiditis. The cytological features found during thyroid fine-needle aspiration include the presence of large multinucleated giant cells or epithelioid granulomas, but the absence of these findings does not exclude the diagnosis of subacute thyroiditis [10,11]. Generally speaking, elevated serum thyroid hormones, a tender enlarged thyroid, and low radioiodine thyroid uptake are characteristic of subacute thyroiditis [12]. Although cases with these typical signs may present little difficulty for a diagnosis of subacute thyroiditis, this disorder does not always present in a classic fashion and may lead to difficulties during diagnosis [5,6]. Sometimes the diagnosis may be less clear, particularly when the primary presenting symptom is a solitary thyroid nodule in conjunction with normal thyroid function, thyroglobulin levels, and a normal ESR [13].

Previously, thyroid nodules have been identified in association with subacute thyroiditis and, in some patients with subacute thyroiditis, only one nodule is present [14,15]. For example, Liel [16] reported a case that presented with the coexistence of subacute thyroiditis and an autonomously functioning thyroid nodule. More often, localized forms of subacute thyroiditis present as painful and tender “cold” thyroid nodules, which disappear following recovery [14]. However, subacute thyroiditis that presents as a painful “hot” nodule is exceedingly rare and has not been reported. In this case, laboratory tests, including white blood cell count, neutrophil percentage, thyroid function, thyroglobin levels, and ESR, were normal and non-diagnostic but the clinical findings (neck pain, thyroid tenderness, and fatigue) led to the consideration of a diagnosis of subacute thyroiditis. Therefore, further work-ups were completed including an ultrasound examination of the neck, thyroid scintigraphy with 99 m-Tc, and fine needle aspiration cytology of the nodule. Ultrasound examination demonstrated a dyshomogeneous and hypoechoic mass in the thyroid, which was characteristic of subacute thyroiditis, and thyroid scintigraphy showed a focal accumulation of radiotracer uptake in the thyroid nodule. The histological features of the nodule were also typical of subacute thyroiditis. Therefore, a diagnosis of localized subacute thyroiditis was given and the patient was prescribed prednisone, which resulted in the disappearance of the hot thyroid nodule. The treatment of subacute thyroiditis is essentially symptomatic and includes non-steroidal anti-inflammatory agents or, occasionally, glucocorticoids if the symptoms are prolonged or severe. In the current case, treatment with steroids resulted in an amelioration of the patient’s symptoms and the disappearance of the thyroid nodule after 2 months.

Based on the course and the clinical presentation of the present case, a diagnosis of subacute thyroiditis could be established. The disappearance of the thyroid nodule following prednisone treatment further confirms the diagnosis of subacute thyroiditis following presentation with a thyroid hot nodule. Here, the appearance of the hot thyroid nodule was unusual in that it did not show the usual pattern of low uptake during radioisotope scanning. This case demonstrates that subacute thyroiditis may present as a solitary painful hot nodule in conjunction with normal thyroid function, thyroglobulin levels, and ESR and should, therefore, be considered in the differential diagnosis of such lesions.

The mechanism of 99 mm-Tc localization in subacute thyroiditis is not known. Tonami et al. [17] reported two cases of subacute thyroiditis in which thyroid scintigrams with 201TI chloride showed increased radionuclide activity in the affected areas but decreased activity in the affected areas following thyroid scintigrams with 99 m-Tc. It was presumed that this is primarily due to increased membrane permeability in the inflammatory lesion without the apparent destruction of the thyroid gland, which is typically indicated by normal thyroglobulin levels and thyroid function in the patient. Therefore, the present case suggests the clinical and pathological heterogeneity of subacute thyroiditis.

In conclusion, this case demonstrates that subacute thyroiditis should be considered as a differential diagnosis following presentation with a solitary painful thyroid hot nodule in conjunction with normal thyroid function, thyroglobulin levels, and ESR. Additionally, this case emphasizes the heterogeneous pattern of thyroid imaging in subacute thyroiditis.

### Consent

Written informed consent was obtained by the patient for the publication of this case report and any accompanying images.

## Competing interests

The authors declare that they have no competing interests.

## Authors’ contributions

LL drafted the manuscript; WX collected all medical reports of the patients and LHK revised the manuscript critically. HB performed ultrasound examination and ZHZ carried out pathological examination. All authors read and approved the final manuscript.

## Pre-publication history

The pre-publication history for this paper can be accessed here:

http://www.biomedcentral.com/1472-6823/14/4/prepub
